# Supplementary material for: Point Prevalence of Complementary or Alternative Medicine Use among Children Attending a Tertiary Care Hospital
Source: Children (Basel). 2023 Jan 10;10(1):132. doi: 10.3390/children10010132 (PMC9856932; doi:10.3390/children10010132)
Supplement: Supplementary file 1 [file children-10-00132-s001.zip › AVR_File S1.pdf]

CAM guide sheet

What is Complementary and Alternative Medicine (CAM)?

CAM is a term used to describe a medical product or practice that is not considered 'standard medical care.' When this is used alongside conventional medicine it is considered **complementary.** When it is used instead of conventional medicine it is considered **alternative.**

What are some examples of CAM?

**Acupuncture**	Fine needles are placed into different parts of the body.
**Aromatherapy**	The use of essential oils through inhalation, vaporisation, bath, shower, massage etc.
**Ayurveda**	Traditional Indian system of healing including diet, supplements, yoga, meditation, and massage.
**Traditional Chinese Medicine**	Many different treatments such as acupuncture, Chinese herbal medicine, moxibustion, cupping, tuina, and tai chi.
**Chiropractic Therapy**	Moving parts of the spine to ease pain.
**Herbal products and dietary supplements**	These include products that are found in nature including vitamins, probiotics, garlic, ginkgo, ginseng.
**Homeopathy**	Involves the use of diluted substances.
**Massage**	This is used to help circulation, ease tension in muscles and decrease stress.
**Osteopathy**	Involves gently moving your bones and muscles to ease pain and help you feel better.
**Rongoā Māori**	This is an important part of health care for many Māori. It is based on a holistic approach to health. It includes physical therapies, spiritual healing and plant-based remedies.
**Prayer**	Prayer is classified as a mind-body intervention. It may provide a sense of control to patients and allow reflection during tough times.

CAM use survey

Do you use any type of CAM for your child? (child named on the consent form)

- ☐ Yes
- ☐ No

If you answered 'No' - would you consider using CAM for your child if a doctor recommended it?

- ☐ Yes
- ☐ Maybe
- ☐ No

If you answered 'Yes' please tell us the following information for each type of CAM your child uses

A. What is the name of the CAM?____

B. Where did you get this CAM (most recent time)

- ☐ Pharmacy
- ☐ Supermarket
- ☐ Specialist shop
- ☐ CAM Specialist
- ☐ Ordered online
- ☐ From a family member
- ☐ From a friend
- ☐ Other__________________________________

C. Where have you got information about this CAM? (Please tick all that apply)

- ☐ Pharmacy
- ☐ Specialist shop
- ☐ CAM specialist
- ☐ GP
- ☐ Hospital specialist
- ☐ Nurse/health professional
- ☐ Book/magazine/newspaper
- ☐ Online
- ☐ Advert
- ☐ Family member
- ☐ Friend
- ☐ Other_______________________________________

D. How much did this CAM cost? (most recent)

- ☐ Free
- ☐ Under $20
- ☐ $20-$50
- ☐ $50-$100
- ☐ $100+

E. How often does your child use this CAM?

- ☐ Daily

- ☐ Weekly
- ☐ Monthly
- ☐ Every 6 months
- ☐ Yearly
- ☐ When needed
- ☐ Other

F. How long has your child been using this CAM?

- ☐ Less than 1 month
- ☐ 1-6 months
- ☐ 6-12 months
- ☐ Longer than 12 months
- ☐ Other

G. Why does your child use this CAM? (Please tick all that apply)

- ☐ Treatment of symptoms
- ☐ Prevention of symptoms
- ☐ To complement conventional or prescription treatment
- ☐ There is a lack of conventional or prescription treatment
- ☐ Worry about side effects from conventional or prescription treatment
- ☐ Lack of confidence in conventional or prescription treatment
- ☐ Knowledge of it working for other people
- ☐ Other ____

H. Have you told your child's medical team treating them in hospital that they use this CAM?

- ☐ Yes
- ☐ No
- ☐ Other

I. Have you seen any benefits from your child using this CAM?

- ☐ No change
- ☐ Improved slightly
- ☐ Improved lots

Other_________________________

J. Have you noticed any side effects from your child using this CAM?

- ☐ No side effects
- ☐ Mild side effects
- ☐ Moderate side effects
- ☐ Severe side effects

K. Does your child use this CAM for a chronic/underlying condition?

- ☐ Yes
- ☐ No

Ki. What condition does your child use this CAM for?

____

<u>CAM Opinion Survey</u>

	Strongly agree	Agree	Neutral	Disagree	Strongly disagree
1. Doctors should be supportive of people using CAM					
2. Doctors should ask patients if they are using CAM					
3. Doctors should know about CAM and be able to give advice					
4. I would only use CAM for me or my child if a Doctor recommended it					
5. CAM do not interfere with prescribed drugs					
6. Enough is known about the effectiveness of CAM					
7. Enough is known about the safety of CAM					
8. Enough is known about the side effects of CAM					
9. There is sufficient information available about CAM					
10. CAM have fewer side effects than prescribed or conventional treatment drugs					
11. CAM is more effective than prescribed or conventional treatment					
12. CAM therapists/practitioners have to be qualified and registered					
13. CAM is used by people who lack confidence in conventional or prescribed treatment					
14. CAM is used by people due to a lack of conventional treatment for an illness or condition					
15. CAM can be used to replace conventional treatment					
16. The cost of CAM puts people off using it					
